# Seasonal malaria chemoprevention in northern Mozambique: a cost-effectiveness analysis

**DOI:** 10.1186/s12936-025-05401-x

**Published:** 2025-05-21

**Authors:** Neide Canana, Ivan Alejandro Pulido Tarquino, Sónia Enosse, Kevin Baker, Maria Rodrigues, Christian Rassi, Akashdeep Singh Chauhan, Chuks Nnaji, Baltazar Candrinho, Elisa M. Maffioli

**Affiliations:** 1 Malaria Consortium, Avenida Elias Lucas Kumato, 118 - Maputo, Mozambique; 2 Malaria Consortium, The Green House, 244-254 Cambridge Heath Road, London, E2 9DA UK; 3 Indian Institute of Public Health-Delhi, Public Health Foundation of India, House No. 60, 4 th Floor, Lane 2, Part of Saidulajab Extension, Near Saket Metro Station Gate No. 2, New Delhi, 110030 India; 4 National Malaria Control Programme, Ministry of Health, 2HJP+86Q, Avenida Eduardo Mondlane, Maputo, Mozambique; 5 Department of Health Management and Policy, School of Public Health, University of Michigan, 1415 Washington Heights, Ann Arbor, MI 48109 USA

**Keywords:** Cost-effectiveness, Malaria, Seasonal malaria chemoprevention (SMC), Mozambique

## Abstract

**Background:**

Malaria is endemic in Mozambique and one of the leading causes of death in children under 5 years old. In 2020 the country adopted the WHO-recommended seasonal malaria chemoprevention (SMC) strategy and delivered the intervention in all 23 districts of Nampula province between January and April 2023. The aim of this study is to estimate the cost-effectiveness of SMC in Nampula, Mozambique.

**Methods:**

Financial cost of implementing SMC were estimated from a limited health care provider perspective for the year 2023 in US$. Data on resource use of the SMC implementation was assessed from Malaria Consortium records. The number of eligible and treated children was collected from surveys after cycle 4. The number of malaria cases, deaths and Disability Adjusted Life-Years (DALYs) averted were estimated based on data from Global Burden of Disease 2019, Malaria Indicator Survey 2018, and National Malaria Control Programme. Incremental cost-effectiveness ratios (ICERs) were estimated, and sensitivity analyses were used to test the robustness of the ICERs.

**Results:**

The total financial cost of SMC implementation in Nampula province in 2023 was estimated to be $7,871,361.72. The study estimated a cost per targeted child of $6.05 and a cost per child who received full 3-day course of sulfadoxine-pyrimethamine in combination with amodiaquine (SPAQ) of $7.92. Furthermore, the cost per household with eligible children visited by a community distributor was $7.65; the cost per child who received day 1 SPAQ was $7.85 and the cost per child who received day 1 SPAQ by community distributor adhering to directly observed treatment was $8.50. In addition, the estimated cost was $93.50 per malaria case averted, $3286.59 per malaria death averted, and $130.16 per DALY averted. The ICERs were robust to a variety of alternative assumptions on costs and benefit estimates. Finally, $1,726,189.63 could have been saved if no ineligible children (60–119 months old) were treated through the programme.

**Conclusions:**

In line with existing evidence from other African countries, SMC is found to be cost-effective in Mozambique. SMC is a beneficial prevention strategy to improve under-five health in the country, at a relatively low-cost.

**Supplementary Information:**

The online version contains supplementary material available at 10.1186/s12936-025-05401-x.

## Background

Global malaria progress has stalled in recent years. Since 2019 several endemic countries have suffered humanitarian and health emergencies, and this has been compounded by disease outbreaks, including the COVID-19 pandemic [1]. As of 2022, there were an estimated 249 million malaria cases, an increase of 5 million compared to the year before [1]. The World Health Organization (WHO) African Region accounted for about 94% of malaria cases globally in 2022, and only four countries—Nigeria (27%), the Democratic Republic of the Congo (12%), Uganda (5%) and Mozambique (4%)—accounted for almost half of all cases globally [1]. While the percentage of total malaria deaths aged under 5 years had declined steadily from 87% in 2000 to 76% in 2015, there has not been much further decline since then, with estimated deaths of 608,000 in 2022 [1].

Mozambique faces a significant malaria burden, with approximately 10.4 million malaria cases reported in 2022 [1]. Malaria accounts for 29% of all deaths and 42% of deaths among children under 5 years in the country, making it the most significant national public health threat [2]. The Mozambique National Malaria Control Programme (NMCP) adopted the “high burden to high impact” approach with its strategic plan for 2017–2022 focusing on reducing the burden of malaria in high endemic areas and sustaining gains in low transmission areas towards elimination [3]. Prior to this, Mozambique has implemented three-yearly universal distribution of long-lasting insecticide-treated bed nets, intermittent preventive treatment in pregnancy, and a horizontal approach to testing, treatment and surveillance as a part of regional initiative MOSAWA [3]. Mozambique had also administered intermittent preventive treatment in infants alongside routine vaccinations [4].

Seasonal malaria chemoprevention (SMC) has been recommended by the WHO since 2012, to prevent malaria in areas where malaria transmission is seasonal [5, 6]. To date, SMC has mainly been implemented in 17 countries of the Sahel region and other seasonal areas of sub-Saharan Africa [1]. SMC provides protection against *Plasmodium falciparum* malaria to at-risk populations during the peak malaria transmission period, which usually corresponds to the rainy season. The WHO originally recommended a monthly course of sulfadoxine-pyrimethamine (SP) in combination with amodiaquine (AQ) to children between 3 and 59 months over four consecutive monthly cycles. Each full course of SPAQ confers protection from malaria for approximately 28 days [7]. The objective of SMC is to maintain therapeutic drug concentrations in the blood throughout the period of greatest risk [7].

The WHO initially recommended the scale up of SMC in the Sahel region only and classified areas eligible for SMC as those in which: (i) over 60% of clinical malaria cases occur within a four-month period, (ii) the clinical attack rate of malaria is greater than 0.1 episodes per child during the transmission season in the target group, and (iii) the resistance to SPAQ has not developed such that its protective efficacy remains above 90% [6, 7]. In 2023, WHO published consolidated guidelines for malaria, which confirm the recommendation to implement SMC in areas of seasonal malaria transmission. The consolidated guidelines no longer define geographic restrictions. They also no longer specify the target age range but recommend that SMC should be given to age groups at high risk of severe malaria [8]. An updated SMC field guide specifies that SPAQ remains the drug regimen of choice and that full courses of SPAQ should be given at 28-day intervals, beginning at the start of the transmission season and continuing for 3–5 cycles, depending on the local context. In fact, depending on the seasonal patterns of malaria transmission, the timing and number of SMC cycles may vary between countries and in different parts of the same country. The field guide confirms SMC eligibility criteria i) and ii) from the original policy recommendation but makes no reference to (iii) [6]. Currently, quality assured SPAQ for SMC is only available for children 3–59 months.

In Mozambique, SMC was initially introduced during 2020–2021 as a component of a pilot investigation in two districts within the Northern Province of Nampula. Eligibility criteria and implementation details are described elsewhere [9, 10]. After the initial implementation, which yielded positive outcomes and demonstrated significant community-level impact, SMC was expanded to include an additional two districts in the subsequent year, thereby extending its reach to a total of four districts between January and April 2022. This subsequent round of implementation was characterized by a more rigorous methodological approach, employing a cluster randomized controlled trial [11] for assessing the effectiveness of SMC. Both phases were conducted by the Ministry of Health (MoH) and the NMCP with support from Malaria Consortium, which in response to the successful outcomes, decided with Malaria Consortium as implementer to scale the programme to the provincial level. Consequently, SMC was rolled out to encompass all 23 districts of Nampula province during the high transmission season, extending from January to April 2023.

There is evidence that SMC is efficacious, safe and cost-effective in the Sahel region [12–14]. While there is also growing evidence that SMC is effective in other areas of Africa where malaria transmission is seasonal [15, 16], evidence of the cost-effectiveness of SMC outside the Sahel region is lacking. This study was undertaken to estimate the cost-effectiveness of implementing SMC in Nampula province of Mozambique for the year 2023. Additional analysis in Appendix A considers the three rounds of SMC implemented between 2020 and 2023 separately. Cost-effectiveness was measured in terms of cost per household with children eligible covered, cost per child receiving day 1 SPAQ, cost per child receiving day 1 SPAQ by directly therapy (DOT), and cost per child receiving the full 3 days course. A cost per malaria case and malaria death averted were also estimated, as well as a cost per Disability Adjusted Life-Years (DALYs) averted. Finally, the study estimated potential savings if SMC would have been provided only to eligible children (3–59 months).

## Methods

### Seasonal malaria chemoprevention (SMC)

**Fig. 1:**
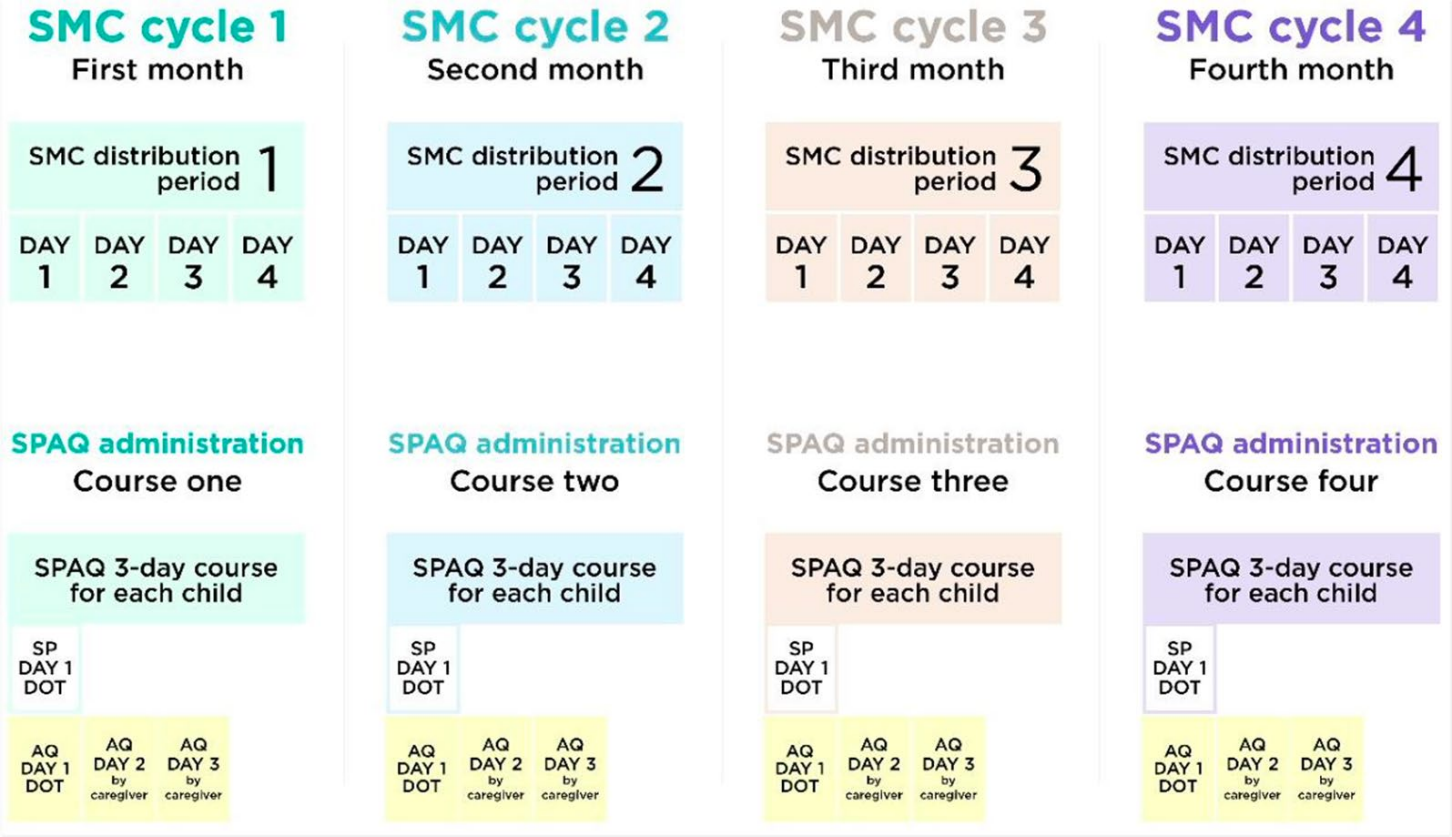
Illustration of schedule for an annual round of SMC in areas with 4 cycles. This has been reproduced by Malaria Consortium (Malaria Consortium, 2021)

In Mozambique, SMC was delivered door-to-door over a period of four days by trained community distributors in each of four monthly cycles during the high transmission season for all children 3–59 months old (Fig. 1). Each monthly SPAQ course consisted of one single dispersible tablet of SP and three daily dispersible tablets of AQ. There were two dosages of SPAQ that were used: a lower dosage for children 3 to < 12 months, and a higher dosage for children 12–59 months. A dose of SP and the first dose of AQ (*day 1 SPAQ*) were administered by or under the supervision of community distributors to ensure that the tablets were correctly dispersed in water and that the child fully ingests all the dispersed tablets without spitting them out or vomiting. This is referred to as directly observed treatment (DOT). Children who vomit or spit out most of the medicine within 30 min were given one replacement dose of SP and AQ by distributors. The remaining two doses of AQ were administered by the caregiver once per day (every 24 h) over the following two days (day 2 AQ and day 3 AQ). Community distributors left a blister pack with the two remaining tablets with caregivers and provided instructions on how to administer and record the doses on the SMC child record card. If a child vomits or spits out the second or third dose of AQ, caregivers were encouraged to visit the nearest health facility or contact the community distributor by mobile phone to receive a replacement dose.

The SMC protocol used in Mozambique specifies that SPAQ should not be administered to children outside the eligible age range of 3–59 months, since for older children the formulations specified above are unlikely to provide sufficient antimalarial drug concentrations in the blood to provide protection throughout the 28-day period of each cycle and they might likely contribute to the development of drug-resistant *P. falciparum* malaria [17]. SMC should also not be administered to children with an acute febrile illness who: test positive for malaria; are severely ill; are unable to take oral medication; are receiving co-trimoxazole prophylaxis; have taken a single dose of either SP or AQ, or any sulfamide-containing medicine during the past 4 weeks; or have a known allergy to either SP or AQ, or a known allergy to sulfamide-containing medicines such as co-trimoxazole. Community distributors are also instructed to refer children with fever to the nearest health facility, where they should be tested for malaria using a rapid diagnostic test (RDT). If the test result is negative, children should be given SP and the first dose of AQ by the health facility worker, giving the remaining two doses of AQ to the caregiver for administration over the following two days. If the test result is positive, they should be treated for malaria as per national treatment guidelines. Determining children's ages accurately is challenging because caregivers often do not know their children's exact ages. Additionally, civil registration and identification systems are underdeveloped, and there is a high prevalence of malnutrition in areas with high malaria risk thus children might look younger than what they are. Even though community distributors are given training on methods to determine a child’s age, the administration of SPAQ to children outside the eligible age range is frequently reported [18–22]. Moreover, community distributors may face pressure from caregivers to administer SPAQ to older children, as SMC is seen as an effective protection from malaria [23]. The community distributors are coordinated and supervised by salaried, facility-based health workers. Distribution teams typically comprise a pair of community distributors, who are assigned a supervisor whose role is to ensure that activities are carried out in compliance with agreed procedures. Teams also go accompanied by community leaders who received a monetary recompense as well.

In Mozambique, the rainy season typically lasts from November or December until end of April, not aligning with the calendar year. A first round of SMC was implemented between November 2020 and February 2021 in two districts in Nampula province (Malema and Mecubúri), targeting 87,551 eligible children, and as part of a pilot study on acceptability, feasibility, and effectiveness of SMC [9]. The second round was conducted between January and April 2022 in 4 districts (Malema, Mecuburi, Muecate and Lalaua), targeting 114,276 eligible children in each cycle [11]. To identify suitable districts for SMC, an SMC suitability ranking was applied to all provinces. Criteria included: (i) seasonality eligible for SMC (60% of rainfall concentrated in 4 months); (ii) mortality (areas of highest under five mortality using BES/SISMA data); (iii) access to care (highest ranking given to areas where access to care was poor); and (iv) treatment seeking behavior (highest ranking given to areas where treatment seeking behavior was poor). An average of these 4 criteria was taken to identify the top 20 suitable districts. From the list of suitable districts, an additional consideration was taken given the importance of implementing SMC in an area where no other new interventions were taking place. Malema and Mecuburi districts in Nampula province were selected as intervention districts for Phase 1 of this pilot project, with Lalaua district serving as a control [9]. For Phase 2, two additional districts (Lalaua, the control district in Phase 1, and Muecate) were added for the purpose of further research studies [11]. A third round was implemented in January-April 2023 scaling up to all 23 districts targeting 1.3 million children [19–22]. A four-cycle round was conducted in early 2024.

### Study design

A cost-effectiveness analysis of SMC implementation in Nampula, Mozambique was conducted. A limited health care provider perspective was used, in which only healthcare costs borne by implementers for the delivery of the intervention (e.g., MoH, Malaria Consortium, or donor agencies) were included. The primary analysis focused on round 3 conducted between January and April 2023 during which SMC was implemented in the entire province of Nampula. Cost data on SMC implementation were retrieved from the records of Malaria Consortium. Measures of benefits were assessed from Malaria Consortium research studies [19–22] as well as from NMCP reports to measure incidence. Data on malaria prevalence and mortality were assessed from available data sources such as the Global Burden of Disease Study [24] and Malaria Indicator Survey [25]. Additional analysis in Appendix A considers the three rounds of SMC implemented between 2020 and 2023 separately.

### Costs

Provider cost of SMC (in 2023 US$) implementation was estimated using ingredients-based approach. Financial records of Malaria Consortium for the period from August 1, 2020 to September 31, 2023 (for all three rounds) were obtained retrospectively and reviewed, but the main analysis mainly focused on the third round of SMC in 2023 (see Appendix A for analysis by round). Direct costs incurred on SMC implementation were categorized in commodities, SMC delivery, operations, human resources, office & equipment, and vehicles. Research and evaluation costs were excluded from this analysis.

The costs incurred on the procurement of commodities, drugs and other supplies, and transportation were categorized under the category of *Commodities*. Under the category of *SMC delivery*, costs of planning, community engagement, training and implementation tools, SPAQ administration, supervision, and technical monitoring and evaluation (M&E) were included. More specifically, *planning* included costs for engagements at country, province and micro-planning levels; *community engagement* included costs of mobilization, communication and advocacy; *training and implementation tools* included costs to train community distributors, their supervisors and community leaders and training tools. In round 3 (across all 4 cycles), 625 training sessions were conducted with a total of 14,620 participants. Costs included: (i) Training materials: Development and printing of training manuals, handouts, and job aids; (ii) Logistics: Venue rental, transportation, and accommodation for participants (where applicable); (iii) Personnel: Trainers'fees, per diems, and travel expenses; (iv) Refreshments: Providing meals and snacks during training sessions. The trainings were conducted at national, provincial, district and implementer levels. See more information in Appendix Table A1. *SPAQ administration* covered costs of administrating the drugs, including wages of community distributors; *supervision* included costs for travel to support supervision and in-process monitoring by Malaria Consortium staff; *technical & M&E* included costs for M&E support by the technical staff. The costs of *Operations* as covering logistics by managers, procurement office activities, visit and travel management and other operational expenses within the country were also categorized. The category of *Human resources* included salaries of administrative, finance, and operations staff, project managers, communication and mobilization staff, data analysts, digital specialists, drivers, additional M&E specialists and security personnel. In round 3, the majority of costs were allocated to logisticians (3 at 67% time effort); finance and management accountants (2 at 45% time effort); country director (1 at 40% time effort); drivers (5 at 100% time effort); the country technical coordinator (1 at 50% time effort); the SMC M&E officer (1 at 100% time effort); the communication manager (1 at 45% time effort); the administration assistant (1 at 100% time effort); other human resources (2 at 40% time effort); the SMC project manager (1 at 100% time effort); and the M&E specialist (1 at 100% time effort). Other human resources included accountant, country mobilization officer, country project manager, data analyst, digital health specialist, digital staff, operations staff, security police vigilantes, senior management staff, staff finance assistant, technical staff and M&E database officer. The category of *Office & equipment* included expenses for office space, digital devices, other equipment, and translation services. Office costs include items such as rent, utilities, cleaning, office supplies, internet, water, and other office-related expenses. Office equipment primarily consists of furniture, with a small portion allocated to office computers. The costs of purchasing computers were included in each round. Since office equipment accounts for only 0.5% of the total costs—making it negligible relative to overall expenses—and no exact details were available on which laptops or furniture were purchased to determine their lifespan, these costs were treated as a one-time expense instead of depreciating them. The category of *Vehicles* covered costs of cars and motorbikes. Opportunity cost of donated goods, volunteer time and productivity losses of volunteers and caregivers during treatment of malaria were not included. All the costs were measured in US$ for the year 2023. In addition to the SMC implementation cost, the savings from reduced case management expenditures due to the decrease in malaria cases resulting from SMC implementation were also estimated. The provider cost of treating uncomplicated and severe malaria were taken from [26].

### Benefits

The number of children (3–59 months) targeted and reached by the programme was obtained from Malaria Consortium coverage reports [19–22]. SMC programme coverage was assessed using end-of-cycle (EoC) surveys and comprehensive end-of-round (EoR) surveys. EoC surveys employing the lot quality assurance sampling (LQAS) methodology were conducted following all but the final SMC cycle, to enable implementing teams to identify areas of low coverage and other issues in SMC delivery. EoC surveys then enabled SMC programme teams to rapidly take corrective actions to improve SMC delivery in subsequent cycles. These surveys were completed within 2 weeks of the completion of the SMC cycle. Comprehensive EoR surveys took place within 8 weeks of completion of the final monthly cycle (i.e., cycle 4). EoR surveys are designed to be representative at the country level to assess programme performance across all monthly cycles in which SMC was delivered.

Based on the data availability from publicly available coverage reports [19–22], the total number of eligible children (3–59 months) was first estimated. Then estimated the following measures were estimated: (1) households with eligible children visited by a community distributor (cycle 4); (2) eligible children who received day 1 SPAQ administered by community distributors; (3) eligible children who received day 1 SPAQ with community distributors observing DOT (among children who had received day 1 SPAQ); (4) eligible children who received a full three-day course of SPAQ (including both day 2 and day 3 AQ, among children who had received day 1 SPAQ); (5) ineligible children 60–119 months (as day 1 SPAQ administered by community distributors) to estimate the potential savings by the implementer. Key indicators (1), (2), (3), (4) were measured through EoC surveys for some, but not all cycles rounds. Measures were available across rounds in Mozambique only for cycle 4, which were used in the analysis. Key indicator (5) was instead measured through the EoR survey.

Furthermore, the number of malaria cases and deaths averted with SMC were estimated. For cases, malaria incidence was estimated by dividing the recorded new cases of malaria for children under 5 years old, as reported from the NMCP, by the population at risk. Following [11], the population at risk were retrieved from the National Census 2017 of which about 16% were children under 5 years old, adjusted by an annual growth factor of 3.5% in Nampula province by the National Institute of Statistics, resulting in a total of 987,056 children at risk. Estimates of SMC efficacy were then used from the research associated with SMC which are available for round 1 and 2 [27, 28] at 82% and 77%, respectively. Since results are not yet available for round 3, a similar efficacy was conservatively used as for round 2, at 77%. A less conservative estimate of 82% efficacy was used in sensitivity analysis. Alternatively malaria prevalence was used for estimating malaria cases averted in the sensitivity analysis. Specifically, a lower bound was used for malaria prevalence in Nampula region for children 3–59 months at 35%, as estimated by GiveWell [29] from the GBD 2019 [24] and adjusted for this sub-population. Other estimates, such as from MIS 2018 [25], mentions a prevalence of 47.9% in Nampula for children 6–59 months.

The number of malaria cases that would have occurred in the case in which children did not receive SMC (counterfactual scenario) was estimated by multiplying malaria incidence by the number of children 3–59 months who received the full three-day course of SPAQ. Estimates of the number of malaria cases averted by SMC were then obtained by multiplying the estimated total number of malaria cases in the counterfactual scenario by 77% (i.e., assuming effectiveness of 77% in round 3 conservatively, with no effect outside this period).Estimates of the number of malaria cases averted by SMC in each round were then obtained by multiplying the estimated total number of malaria cases in the counterfactual scenario by 82% and 77% (i.e., assuming effectiveness of 82% in round 1 and conservatively 77% in round 2 and 3, with no effect outside this period).

GBD 2019 and regional data of Mozambique were used to estimate a malaria mortality rate of 3.05 per 1000 children under 5 per year. Malaria deaths were derived in the counterfactual scenario (no SMC) and with SMC for the population of children 3–59 months who were treated in that cycle, assuming a reduction in mortality of 79% due to SMC. Similar calculations conducted by GiveWell [29], but excluding the deaths under 3 months, estimated a malaria mortality rate of 2.8 per 1000 children 3–59 months. Alternative estimates of reduction in mortality due to SMC are estimated to be 66% in Mali [30] and 42.4% in Burkina Faso and 56.6% in the Gambia [12]. No results are available yet for Mozambique. These alternative assumptions were used in the sensitivity analysis.

Finally, DALYs were estimated by summing up the years of life lost to due to premature mortality (YLLs) and the years lived with a disability (YLDs). YLL was calculated as “*number of deaths averted X standard life expectancy*”, with the life expectancy taken as gender and age specific from GDB 2019 [24]. YLD was calculated as “*number of cases averted X duration of disability X disability weight*”. The duration of an uncomplicated malaria episodes was estimated at 7 days, expressed in years (7/365) and for a severe episode, 30 days, expressed in years (30/365) following the GBD 2019 [24] (disability weight of 0.006 for uncomplicated malaria and 0.133 for severe cases, GBD 2019). To estimate the proportion of severe cases out of total malaria cases in Nampula province, data from NMCP reports were used indicating severe cases constituted approximately 1% of all malaria cases (whether uncomplicated or severe) among the general population.

### Cost-effectiveness

The SMC intervention was considered as an addition to the status quo. Thus, incremental cost-effectiveness ratios (ICER) of SMC were calculated as the incremental costs divided by the incremental effects, i.e., the additional effect (e.g., cases averted, or deaths averted). Cost-effectiveness ratios were calculated by dividing the total cost of the SMC intervention by the different measures of benefits for round 3 in 2023.

The following key measures of cost-effectiveness were reported:

1. Cost per household with eligible child visited by community distributor
2. Cost per eligible child who received day 1 SPAQ in terms of children who received day 1 SPAQ during cycle 4 and in the three previous monthly cycles
3. Cost per eligible child who received day 1 SPAQ by community distributors by directly observed therapy (DOT) or supervision during cycle 4 (that is, among eligible children who received day 1 SPAQ in that cycle as denominator)
4. Cost per eligible child who received a full three-day course of SPAQ (including day 2 and day 3 AQ) during cycle 4 (that is, among eligible children who received day 1 SPAQ in that cycle as denominator)
5. Cost per malaria case averted
6. Cost per malaria death averted
7. Cost per DALY averted

Given the measure of cost per eligible child who received day 1 SPAQ, the potential cost savings for implementers, if ineligible (older, above 59 months) children were not provided with SMC, were also estimated, by multiplying the cost per eligible child who received day 1 SPAQ by the number of ineligible children (60–119 months) who received day 1 SPAQ.

### Sensitivity analysis

Sensitivity analyses were undertaken to assess the robustness of cost effectiveness estimates, by varying key cost and effectiveness indicators, which had some degree of uncertainty. First, the costs were adjusted by including savings from the treatment of malaria cases averted with SMC. Second, key benefit indicators were varied by using low and high extreme values of 95% Confidence Intervals, including the proportion of households with eligible children visited by a community distributor, the proportion of eligible children (3–59 months) who received day 1 SPAQ, the proportion of eligible children (3–59 months) who received day 1 SPAQ by community distributors adhering to DOT (among those who received day 1 SPAQ) and the proportion of eligible children (3–59 months) who received the full three-day course of SPAQ among those who received day 1 SPAQ. Third, the protective efficacy of SMC for round 3 was varied from a conservative estimate of 77% to a higher estimate of 82% observed in round 1.

Fourth, malaria prevalence estimates rather than incidence for estimating malaria cases averted were used. A lower bound for malaria prevalence in Nampula region for children 3–59 months at 35% was used, as estimated by GiveWell [29] from the GBD 2019 [24] and an alternative higher prevalence estimate of 47.9% from MIS 2018 [25] for children aged 6–59 months. Fifth, alternatively a malaria mortality rate of 2.8 per 1000 children was assumed, as estimated by GiveWell [29]. Finally, the average reduction in mortality (55%) due to SMC was calculated from existing studies [12, 30] and the cost-effectiveness was estimated in the sensitivity analysis.

## Results

### Costs

Table 1 shows the total costs, aggregated by categories as described in Methods section. A total of $7,871,361.72 million were spent by Malaria Consortium to deliver SMC in the entire province of Nampula. Most of the costs (68.55%) were attributed to SMC delivery, especially training and implementation tools (23.89%), technical & M&E activities (16.89%), and SPAQ administration (14.27%). Another 25.47% were spent on commodities, mainly drugs and freight. A smaller proportion of the resources were spent on human resources (2.97%), office & equipment (1.58%), other operations (0.38%) or vehicles (0.05%). Appendix Table A2 describes these costs at a more granular level and by rounds.

**Table 1:** Total costs of SMC in 2023

	Costs (US$)	Costs (%)
Commodities	$ 2,004,854.90	25.47
SMC delivery		68.55
Planning	$ 150,053.43	1.91
Community engagement	$ 59,793.79	0.76
Training & implementation tools	$ 1,880,172.07	23.89
SPAQ administration	$ 1,123,294.30	14.27
Supervision	$ 852,697.12	10.83
Technical and M&E	$ 1,329,535.69	16.89
Operations	$ 30,137.85	0.38
Human Resources	$ 312,430.74	3.97
Office & equipment	$ 124,654.64	1.58
Vehicles	$ 3,737.20	0.05
Total costs	$ 7,871,361.72	100.00

### Benefits

As shown in Table 2, a total of 1,300,000 children (3–59 months old) were targeted by SMC in 2023 in Nampula province. On average, 79.20% of households with eligible children reported to have been visited by a community distributor during survey data collection after cycle 4, and 77.17% of eligible children received day 1 SPAQ. Among those who received day 1 SPAQ, 99.08% completed the full three-day course of SPAQ. Community distributors adhered to DOT for 92.3% of eligible children among those who received day 1 SPAQ by community distributors during home visits.

**Table 2:** Measures of cost-effectiveness

Costs	
Costs	$ 7,871,361.72
Benefits	
Total targeted children (3–59 mo)	1,300,000
Households with eligible children (3–59 mo) visited by a community distributor	1,029,600
Eligible children (3–59 mo) who received day 1 SPAQ	1,003,210
Eligible children (3–59 mo) who received day 1 SPAQ by community distributors adhering to DOT, among those who received day 1 SPAQ	925,963
Eligible children (3–59 mo) who received a full three-day course of SPAQ, among those who received day 1 SPAQ	993,980
Malaria cases averted	84,190
Malaria deaths averted	2395
DALYs	60,476
Cost-effectiveness	
Cost per targeted child	$ 6.05
Cost per household with eligible children visited by a community distributor	$ 7.65
Cost per child who received day 1 SPAQ	$ 7.85
Cost per child who received day 1 SPAQ by community distributors adhering to DOT, among those who received day 1 SPAQ	$ 8.50
Cost per child who received full 3-day course SPAQ, among those who received day 1 SPAQ	$ 7.92
Cost per malaria case averted	$ 93.50
Cost per malaria death averted	$ 3286.59
Cost per DALY averted	$ 130.16
Estimated savings	
Proportion of ineligible children (60–119 mo)	21.93%
Total ineligible children (60–119 mo) who received day 1 SPAQ	220,004
Total spent on ineligible children as cost per child who received day 1 SPAQ	$ 1,726,189.63

SMC averted a total of 84,190 malaria cases, 2395 malaria deaths and 60,476 DALYs on the sample of fully treated children (3–59 months). The proportion of ineligible children (aged 60–119 months old) who received day 1 SPAQ (after cycle 4) was 21.9% in 2023. Consequently, an estimated 220,004 children aged between 60 and 119 months received SPAQ even if they were not eligible. Appendix Table A3 describes these benefits by round.

### Cost-effectiveness

Table 2 shows that SMC is cost-effective with costs ranging from $6.05 per targeted child to $8.50 per child who received day 1 SPAQ by community distributors adhering to DOT, among those who received day 1 SPAQ. The cost per malaria case averted was $93.50, the cost per malaria death averted was $3,286.59, and the cost per DALY averted was $130.16. Lastly, the potential savings were estimated at about $1.7 million if only eligible children would have received day 1 SPAQ. Appendix Table A3 shows similar measures of cost-effectiveness, by round.

### Sensitivity analysis

Table 3 shows the robustness of ICERs to different alternative assumptions. ICERs remain robust when adjusting for lower and higher rates estimates based on 95% Confidence Intervals for key indicators of cost per child visited and treated. Estimated cost per malaria case averted remained robust (at $87.79) when a higher efficacy value of 82% for round 3 was used. Using malaria prevalence instead of incidence increased the cost-effectiveness from a cost per malaria case averted of $93.50 to $29.38 (assuming 25% prevalence as per GiveWell [29]) and to $21.47 (assuming 47.9% prevalence as per MIS 2018 [25]). In terms of malaria deaths averted, assuming a malaria mortality rate of 2.8 per 1000 children aged 3–59 months, as estimated by GiveWell [29], resulted in a higher cost per malaria death averted of $3607.77. Assuming a lower efficacy of SMC in reducing malaria mortality among children (55%), led to higher cost per malaria death averted of $4720.73. Appendix Table A3 shows similar robustness of ICERs, by rounds.

**Table 3:** Sensitivity analysis

(1) Including savings in case management	
Cost per targeted child	$ 5.76
Cost per household with eligible children visited by a community distributor	$ 7.27
Cost per child who received day 1 SPAQ	$ 7.46
Cost per child who received day 1 SPAQ by community distributors adhering to DOT, among those who received day 1 SPAQ	$ 8.09
Cost per child who received full 3-day course SPAQ, among those who received day 1 SPAQ	$ 7.53
Cost per malaria case averted	$ 88.93
Cost per malaria death averted	$ 3126.21
Cost per DALY	$ 123.80
(2) Key benefits indicators (low range, CI 95%)	
Cost per household with eligible children visited by a community distributor	$ 8.32
Cost per child who received day 1 SPAQ	$ 8.55
Cost per child who received day 1 SPAQ by community distributors adhering to DOT, among those who received day 1 SPAQ	$ 8.87
Cost per child who received full 3-day course SPAQ, among those who received day 1 SPAQ	$ 7.99
(3) Key benefits indicators (high range, CI 95%)	
Cost per household with eligible children visited by a community distributor	$ 7.17
Cost per child who received day 1 SPAQ	$ 7.34
Cost per child who received day 1 SPAQ by community distributors adhering to DOT, among those who received day 1 SPAQ	$ 8.27
Cost per child who received full 3-day course SPAQ, among those who received day 1 SPAQ	$ 7.88
(4) Rate of reduction in malaria cases at 82% (round 3)	
Cost per malaria case averted	$ 87.79
(5) Malaria prevalence at 35%	
Cost per malaria case averted	$ 29.38
(6) Malaria prevalence at 47.9%	
Cost per malaria case averted	$ 21.47
(7) Mortality rate from malaria for 3–59-month-olds at 2.8 per 1000	
Cost per malaria death averted	$ 3607.77
(8) Rate of reduction in malaria deaths at 55%	
Cost per malaria death averted	$ 4720.73

## Discussion

Past evidence suggested that SMC is efficacious and safe. Meremikwu et al*.* [31] found that SMC could prevent about 75% of malaria episodes in children including severe episodes and deaths in West Africa. A randomized clinical trial in Ghana demonstrated that SMC reduced malaria incidence by 40% in the rainy season [32]. Milligan et al*.* [33] found that the scale-up of SMC in seven Sahelian countries reduced the number of confirmed malaria cases at outpatient clinics each year in the range of 25% to 59%. Cairns et al*.* [34] found that children who received SMC were 88% less likely to develop malaria over 28 days than those who had not received SMC across Burkina Faso, Chad, The Gambia, Mali and Nigeria. A meta-analysis of SMC studies, where SPAQ administered once a month during peak malaria transmission season to children under 5 years showed a protective effect of 82%, and a 57% protective effect against all-cause mortality [35]. Another study in Mali found that SMC was associated with a reduction in hospital admissions and all-cause mortality [30]. There is growing evidence that SMC is effective in real-world conditions, including studies in Burkina Faso, Mali and Senegal [36–39]. There is further evidence that SMC can be delivered at scale, achieving high coverage [13, 40].

As of 2023, the WHO provided new guidance on SMC, and, among other changes, removed geographical restrictions on the use of SMC [6, 8], opening up the possibility to test SMC in areas where malaria transmission is seasonal but parasite resistance to SPAQ is high. Resistance to SP, in particular, is widespread in East and Southern Africa [41, 42]. While it has been suggested that SP may retain its protective effect even in areas where resistance is high [43, 44], the relation between the degree of resistance and the effectiveness of SMC has not yet been clearly defined [45], prompting a series of research studies on expanding SMC to areas outside the Sahel region. A recent study in South Sudan reported malaria case load reduction despite the high level of resistance [46]. Other studies in Uganda [15] and South Sudan [16] similarly suggest that SMC is effective in preventive malaria cases during the high transmission season, despite high parasite resistance. There are also on-going studies that aim to test the feasibility and effectiveness of SMC, as well as the protective efficacy of SPAQ, including in Mozambique [11].

Similarly, there exists some evidence on cost-effectiveness from the Sahel region, but it is non-existent from Eastern and Southern Africa, where SP drug resistance is common. Therefore, it is important to study the cost-effectiveness of SMC outside the Sahel to better shed light on its scalability in these contexts as well. In line with existing evidence from other African countries, the findings highlight that SMC is cost-effective in Mozambique, suggesting it is a beneficial prevention strategy to improve under-five health in the country, at a relatively low-cost. In addition, these findings indicate that SMC was more cost-effectiveness during round 3 of its implementation, suggesting economies of scale when SMC was extended to the entire province of Nampula.

These cost-effectiveness estimates are lower or in the ballpark of what existing studies report. A recent meta-analysis [47] of 17 studies from sub-Saharan Africa between 1992 and 2021 found ICERs ranging from $19 to 128 (2020) per malaria case averted and of $3938 per malaria death averted. The study also estimated a cost per dose administered ranging from $0.03 to 1.83, with an average of $0.52 (95% CI 0.29–0.75) per dose per child. The median cost child for full SMC treatment was estimated at $4.32. Similarly, combined evidence from an observational study in Burkina Faso, Chad, The Gambia, Guinea, Mali, Niger, and Nigeria [12] estimated a weighted average economic cost of administering four monthly SMC treatments of $3.63 per child. The closest research conducted in Mozambique [48] is outdated and estimated the cost-effectiveness of malaria intermittent preventive treatment in infants using sulfadoxine-pyrimethamine (SP) in Manhiça. The cost per clinical episode of malaria averted was $4.73 (range: $ 1.7–30.3) while the cost per death averted was $301.1 (range: $95.6–2498.4). The results are also in line with a similar exercise conducted by GiveWell [29], for countries they fund, which estimated a cost per child fully treated of $6.48 and a cost per under-five death averted of $4,054 in Mozambique. In terms of cost per DALYs averted, the estimates are also in line with existing evidence from Mali (US $144 per DALY averted [49]) and for seven countries in the Sahel subregion (a cost per DALY averted ranging from $18.66 in Niger to $78.91 in The Gambia [14]). More importantly, the estimated cost of $130.16 per DALY averted is below the WHO threshold for “highly cost-effective” interventions [50]—those with a cost per DALY less than one time GDP per capita (GDP per capita of Mozambique was $558.3 in 2022, [51]).

This study is not without limitations. First, the analysis adopted a limited health care provider perspective, focusing solely on healthcare costs borne by implementers for delivering the intervention. However, the sensitivity analysis incorporated health system savings from the management of malaria cases averted due to SMC. Additionally, the cost-effectiveness analysis did not account for economic costs such as donated items, volunteer time, and other indirect costs as well as those costs associated with households accessing malaria treatment (e.g., reduction in time spent for health care and testing and treatment expenditures), thus under-estimating the total resources used by SMC. Second, the assessment of SMC benefits was restricted to morbidity and mortality among the population of eligible children receiving SMC during the high transmission season, as estimated by Malaria Consortium’s research [27, 28]. Yet, it is possible that there are positive benefit spillovers for other children in the community not directly treated with SMC, potentially resulting in reduced malaria transmission at the community level over a longer period. Thus, the overall impacts of SMC on 12 months would be different than during the high transmission season. Third, there is some uncertainty regarding the assumptions used for the cost-effectiveness analysis, particularly concerning morbidity and mortality, e.g., incidence data taken from NMCP may underestimate the number of new malaria cases at any point in time [52], and the estimates used for the initial two rounds of SMC are not district-specific, even though only 2 and 4 districts in Nampula province were covered, respectively. Nevertheless, the sensitivity analysis demonstrated robustness of the results by using malaria prevalence and to alternative mortality assumptions. Despite these weaknesses, this is the first cost-effectiveness analysis conducted in Mozambique on the implementation of SMC.

## Conclusion

Despite the implementation of different prevention strategies in Mozambique, the malaria burden remains high, and measures are still necessary to improve children’s health in the country. SMC has been identified as an effective strategy in areas with seasonal malaria transmission. However, there exists limited evidence of the cost-effectiveness of SMC from East and Southern Africa, where parasite resistance to SP is widespread. This study addressed this gap by describing the cost-effectiveness of SMC in Mozambique, where, since 2020, three rounds of SMC have been implemented and the entire province of Nampula has been covered. The analysis, from a limited health care provider perspective, shows that SMC can improve children health in a cost-effective way. While the MoH is refining its Malaria Strategic Plan 2023–2027, which may take some of these results in consideration, additional research and analysis are required to further elucidate strategies to reduce the costs associated with SMC. For example, transitioning from a door-to-door delivery model to a health center-based distribution approach could be considered as part of the strategic planning of public health programmes in the country.

## Supplementary Information

Supplementary material 1.

## Data Availability

Data are available upon request from Malaria Consortium.
