# Supplementary material for: Seasonal malaria chemoprevention in northern Mozambique: a cost-effectiveness analysis
Source: Malar J. 2025 May 21;24:159. doi: 10.1186/s12936-025-05401-x (PMC12096524; doi:10.1186/s12936-025-05401-x)
Supplement: Supplementary file 1 — Supplementary material 1. [file 12936_2025_5401_MOESM1_ESM.docx]

**Seasonal malaria chemoprevention in northern Mozambique:**

**a cost-effectiveness analysis**

**Neide Canana, Ivan Alejandro Pulido Tarquino, Sónia Enosse, Kevin Baker, Maria Rodrigues, Christian Rassi, Akashdeep Singh Chauhan, Chuks Nnaji, Baltazar Candrinho, and Elisa M Maffioli**

**Supplementary Material**

**Table A1: Trainings conducted as part of SMC (Round 3)**

**Cost-Effectiveness Analysis by round**

This analysis considers the three rounds of SMC implemented between 2020 and 2023 separately.

Compared to the main analysis for round 3, note that in terms of costs, we converted all costs for rounds 1 and 2 to 2023 US$ using a rate of 3% (and of 5% in the sensitivity analysis as suggested by Haacker et al. 2020 (Appendix Table A3). In terms of benefits, estimates of the number of malaria cases averted by SMC in each round were then obtained by multiplying the estimated total number of malaria cases in the counterfactual scenario by 18% and 23% (i.e., assuming effectiveness of 82% in round 1 and conservatively 77% in round 2 and 3, with no effect outside this period).

Appendix Table A2 reports the costs for round 1 (2 districts in 2020-2021) and round 2 (4 districts in 2022) in addition to the round 3. Most of the total costs (73.67%) were for round 3, as expected due to the extension to all 23 districts of Nampula. Most costs (64.81%) were for SMC delivery, especially training and implementation tools (22.16%), Technical & M&E (14.74%), and SPAQ administration (13.64%). Another 23.56% were for commodities, mainly drugs and freight. Less resources were spent for human resources (6.99%), office & equipment (2.55%), vehicles (1.32%) or other operations (0.77%). It is worth to highlight that, while the costs of commodities, training, and implementation tools, SPAQ administration, supervision, technical and M&E increased proportionally with the extension to additional districts over the three rounds, the same is not true for other categories of costs. For example, the planning and community engagement costs almost tripled from round 1 to round 2 and remained similar in round 3, suggesting, not surprisingly, at least an initial higher fixed costs to get the program planned. In addition, the costs of human resources and office & equipment increased over the rounds, but not proportionally to the geographical extension. Finally, the costs of operations and vehicles were not increasing, suggesting some costs were up fronted during the first round.

In terms of benefits, as shown in Appendix Table A3, a total of 1,501,827 children (3-59 months old) were targeted by SMC in Mozambique between 2020 and 2023, out of whom 87,551 in round 1 (2 districts), 114,276 in round 2 (4 districts) and about 1,300,000 in round 3 (all 23 districts) in Nampula province. On average 85.5% of households with eligible children reported to have been visited by a community distributor, during survey data collection after cycle 4. However, this proportion decreased from approximately 89.3% to 79.2% from round 1 to 3. There was also a similar decrease over rounds for the proportions of eligible children who received day 1 SPAQ (from 85.8% to 77.17%). The average over the rounds was 81.2%. Instead, the proportion of eligible children who received a full three-day course of SPAQ among those who received day 1 SPAQ was high at least 98% in each single round, increasing over time and averaging 98.5% across rounds. Finally, the proportion of eligible children who received day 1 SPAQ by community distributors adhering to DOT among those who received day 1 SPAQ by community distributors during home visits was estimated at 95.4% across rounds, decreasing slightly from 96.1% and 97.8% in round 1 and 2, respectively, to 92.3% in round 3. We also estimated that the three rounds of SMC averted a total of 103,931 malaria cases, 2,894 malaria deaths on the sample of children (3-59 months) fully treated, and 73,075 DALYs. The proportion of ineligible children defined as 60 –119 months old who received day 1 SPAQ (after cycle 4) was 18.6% across rounds, increasing from 15.3% in round 1 to 18.5% to 21.9% in round 3. Thus, A total of 226,604 children between 60 and 119 months were estimated to have received SPAQ even if not eligible, over the study period.

**Table A2. Total costs by rounds, by sub-categories**

*Notes: ** Malaria Consortium is unable to publicly disclose disaggregated salary costs by cadre; therefore, only the total costs for human resources are reported.

Appendix Table A3 shows that SMC is quite cost-effective as an intervention with costs ranging from $7.19 per targeted child to $34.27 per treated child, across rounds. Cost-effectiveness is lower in round 2, than in round 1. It is particularly high in round 3, suggesting some economies of scale. We also find a cost per malaria case averted of $34.27 and a cost per malaria death averted of $3,916.46 across rounds. The cost effectiveness was particularly low in round 3 at $31.17 per malaria case averted and $3,486.74 per malaria death averted. Lastly, we estimate the potential savings to be about $2.1 million for all rounds of the implementation if only eligible children would be receiving day 1 SPAQ.

Appendix Table A4 shows similar robustness of ICERs, by rounds.

**Table A3: Measures of cost-effectiveness, by round**

|  | Round1 | Round2 | Round3 | All Rounds |
| --- | --- | --- | --- | --- |
|  | Nov 2020- Feb 2021 | Jan-April 2022 | Jan-April 2023 | 2020-2023 |
| **COSTS** | | | | |
| Costs | $ 924,916.12 | $ 1,887,062.19 | $ 7,871,361.72 | $ 10,683,340.03 |
| Costs (inflation, 3%)* | $ 981,243.51 | $ 1,943,674.06 | $ 7,871,361.72 | $ 10,796,279.29 |
| Costs (inflation, 5%) | $ 1,019,720.02 | $ 1,981,415.30 | $ 7,871,361.72 | $ 10,872,497.04 |
| **BENEFITS** | | | | |
| Total targeted children (3-59mo) | 87,551 | 114,276 | 1,300,000 | 1,501,827 |
| Households with eligible children (3-59mo) visited by a community distributor | 78,183 | 100,563 | 1,029,600 | 1,284,062 |
| Eligible children (3–59mo) who received day 1 SPAQ | 75,119 | 92,221 | 1,003,210 | 1,219,834 |
| Eligible children (3–59mo) who received day 1 SPAQ by community distributors adhering to DOT, among those who received day 1 SPAQ | 72,189 | 90,192 | 925,963 | 1,163,722 |
| Eligible children (3–59mo) who received a full three-day course of SPAQ, among those who received day 1 SPAQ | 73,842 | 90,376 | 993,980 | 1,201,049 |
| Malaria cases averted | 6,661 | 7,655 | 84,190 | 103,931 |
| Malaria deaths averted | 178 | 218 | 2,395 | 2,894 |
| DALYs | 4,493 | 5,499 | 60,476 | 73,075 |
| **COST-EFFECTIVENESS** | | | | |
| Cost per targeted child | $ 11.21 | $ 17.01 | $ 6.05 | $ 7.19 |
| Cost per household with eligible children visited by a community distributor | $ 12.55 | $ 19.33 | $ 7.65 | $ 8.41 |
| Cost per child who received day 1 SPAQ | $ 13.06 | $ 21.08 | $ 7.85 | $ 8.85 |
| Cost per child who received day 1 SPAQ by community distributors adhering to DOT, among those who received day 1 SPAQ | $ 13.59 | $ 21.55 | $ 8.50 | $ 9.28 |
| Cost per child who received full 3-day course SPAQ, among those who received day 1 SPAQ | $ 13.29 | $ 21.51 | $ 7.92 | $ 8.99 |
| Cost per malaria case averted | $ 147.32 | $ 253.91 | $ 93.50 | $ 103.88 |
| Cost per malaria death averted | $ 5,515.03 | $ 8,925.69 | $ 3,286.59 | $ 3,730.67 |
| Cost per DALY averted | $ 218.40 | $ 353.48 | $ 130.16 | $ 147.74 |
| **ESTIMATED SAVINGS** |  |  |  |  |
| Proportion of ineligible children (60-119mo) | 15.3% | 18.5% | 21.93% | 18.6% |
| Total ineligible children (60 –119mo) who received day 1 SPAQ | 11,493 | 17,061 | 220,004 | 226,604 |
| Total spent on ineligible children as cost per child who received day 1 SPAQ | $ 150,130.26 | $ 359,579.70 | $ 1,726,189.63 | $ 2,005,588.82 |

Notes: * Costs are as of 2023. Costs for rounds 1 and 2 were inflated to the corresponding costs in 2023.

**Table A4: Sensitivity analysis, by round**

|  | Round1 | Round2 | Round3 | All Rounds |
| --- | --- | --- | --- | --- |
|  | Nov 2020- Feb 2021 | Jan-April 2022 | Jan-April 2023 | 2020-2023 |
| **(1) Including savings in case management** |  |  |  |  |
| Cost per targeted child | $ 10.22 | $ 16.21 | $ 5.76 | $ 6.80 |
| Cost per household with eligible children visited by a community distributor | $ 11.44 | $ 18.42 | $ 7.27 | $ 7.95 |
| Cost per child who received day 1 SPAQ | $ 11.91 | $ 20.08 | $ 7.46 | $ 8.37 |
| Cost per child who received day 1 SPAQ by community distributors adhering to DOT, among those who received day 1 SPAQ | $ 12.39 | $ 20.54 | $ 8.09 | $ 8.77 |
| Cost per child who received full 3-day course SPAQ, among those who received day 1 SPAQ | $ 12.11 | $ 20.49 | $ 7.53 | $ 8.50 |
| Cost per malaria case averted | $ 134.30 | $ 241.96 | $ 88.93 | $ 98.23 |
| Cost per malaria death averted | $ 5,027.66 | $ 8,505.35 | $ 3,126.21 | $ 3,527.80 |
| Cost per DALY | $ 199.10 | $ 336.83 | $ 123.80 | $ 139.71 |
| **(2) Key benefits indicators (low range, CI 95%)** |  |  |  |  |
| Cost per household with eligible children visited by a community distributor | $ 13.06 | $ 19.73 | $ 8.32 | $ 8.81 |
| Cost per child who received day 1 SPAQ | $ 13.65 | $ 21.69 | $ 8.55 | $ 9.32 |
| Cost per child who received day 1 SPAQ by community distributors adhering to DOT, among those who received day 1 SPAQ | $ 13.94 | $ 21.73 | $ 8.87 | $ 9.51 |
| Cost per child who received full 3-day course SPAQ, among those who received day 1 SPAQ | $ 13.40 | $ 21.68 | $ 7.99 | $ 9.06 |
| **(3) Key benefits indicators (high range, CI 95%)** |  |  |  |  |
| Cost per household with eligible children visited by a community distributor | $ 12.18 | $ 18.96 | $ 7.17 | $ 8.10 |
| Cost per child who received day 1 SPAQ | $ 12.61 | $ 20.52 | $ 7.34 | $ 8.48 |
| Cost per child who received day 1 SPAQ by community distributors adhering to DOT, among those who received day 1 SPAQ | $ 13.38 | $ 21.35 | $ 8.27 | $ 9.12 |
| Cost per child who received full 3-day course SPAQ, among those who received day 1 SPAQ | $ 13.62 | $ 21.72 | $ 7.88 | $ 8.97 |
| **(4) Rate of reduction in malaria cases at 82% (round 3)** |  |  |  |  |
| Cost per malaria case averted | $ 147.32 | $ 253.91 | $ 87.79 | $ 101.72 |
| **(5) Malaria prevalence at 35%** |  |  |  |  |
| Cost per malaria case averted | $ 46.30 | $ 79.80 | $ 29.38 | $ 32.65 |
| **(6) Malaria prevalence at 47.9%** |  |  |  |  |
| Cost per malaria case averted | $ 33.83 | $ 58.31 | $ 21.47 | $ 23.86 |
| **(7) Mortality rate from malaria for 3–59-month-olds at 2.8 per 1,000** |  |  |  |  |
| Cost per malaria death averted | $ 6,053.99 | $ 9,797.96 | $ 3,607.77 | $ 4,095.25 |
| **(8) Rate of reduction in malaria deaths at 55%** |  |  |  |  |
| Cost per malaria death averted | $ 7,921.59 | $ 12,820.54 | $ 4,720.73 | $ 5,358.60 |
| **(9) Inflation rate (5%)*** |  |  |  |  |
| Cost per targeted child | $ 11.65 | $ 17.34 | $ 6.05 | $ 7.24 |
| Cost per household with eligible children visited by a community distributor | $ 13.04 | $ 19.70 | $ 7.65 | $ 8.47 |
| Cost per child who received day 1 SPAQ | $ 13.57 | $ 21.49 | $ 7.85 | $ 8.91 |
| Cost per child who received day 1 SPAQ by community distributors adhering to DOT, among those who received day 1 SPAQ | $ 14.13 | $ 21.97 | $ 8.50 | $ 9.34 |
| Cost per child who received full 3-day course SPAQ, among those who received day 1 SPAQ | $ 13.81 | $ 21.92 | $ 7.92 | $ 9.05 |
| Cost per malaria case averted | $ 153.10 | $ 258.84 | $ 93.50 | $ 104.61 |
| Cost per malaria death averted | $ 5,731.29 | $ 9,099.01 | $ 3,286.59 | $ 3,757.01 |

Notes: * Costs are as of 2023. Costs for rounds 1 and 2 were inflated to the corresponding costs in 2023.
